# Increasing the Oxidation Stability and Shelf‐Life Quality of Hazelnuts Using a New Peeling Technique

**DOI:** 10.1111/1750-3841.70384

**Published:** 2025-07-03

**Authors:** Sümeyye Şahin, Yeşim Aydın

**Affiliations:** ^1^ Department of Food Engineering Faculty of Agriculture Ordu University Ordu Türkiye; ^2^ Department of Food Engineering Institute of Science Ordu University Ordu Türkiye

**Keywords:** hazelnut peeling method, hexanal, oleosomes, oxidation stability, peroxide value, volatile compounds

## Abstract

**ABSTRACT:**

Hazelnuts without skin are preferred by consumers. Traditionally, the skin is peeled off by the roasting technique (RT). However, RT causes undesirable reactions such as lipid oxidation. Therefore, pressurized water technique (PWT) was developed as an alternative hazelnut peeling method. In this study, the effect of PWT on quality parameters including chemical composition, ultrastructure, peroxide value (PV), free fatty acidity (FFA), total antioxidant capacity (TAC), total phenolic content (TPC), total aflatoxin content (AFC), and volatile compounds of hazelnut samples during long‐term storage (12 months) was evaluated and compared with RT. Generally, FFA, TAC, AFC, and fatty acid contents in hazelnuts were not affected by peeling methods. Before storage, hazelnuts peeled by PWT exhibited slightly higher TPC and slightly lower protein and oil contents than hazelnuts peeled by RT. Additionally, no peroxide formation was detected in hazelnuts peeled by PWT before storage. Nevertheless, after storage, hazelnuts peeled by RT showed 8.2 times higher PV compared to hazelnuts peeled by PWT. Unlike hazelnuts peeled by PWT before storage, a total of 17 different volatile compounds, mostly lipid oxidation products, were detected in hazelnuts peeled by RT. Hexanal, an indicator for secondary oxidation, in hazelnuts peeled by RT before storage was determined to be 1.9 times higher than hazelnuts peeled by PWT. More burst oleosomes in microstructure of hazelnuts peeled by RT were observed by SEM. These results indicate that PWT demonstrated more oxidation stability and shelf‐life quality in hazelnuts compared to RT. Therefore, among these peeling methods, PWT can be recommended for safe food production.

**Practical Application:**

While the roasting process is used to peel the hazelnut skin, lipid oxidation formation is inevitable during this process. Lipid oxidation products not only deteriorate the product quality but also pose a health risk. The newly developed, pressurized water peeling technique increases the lipid oxidation stability in hazelnuts. Increasing oxidation stability by the pressurized water peeling technique ensures that the quality of the hazelnut is preserved, and its shelf life is increased.

## 1 Introduction

The World Health Organization (WHO) has reported that a diet including nuts supports the prevention of malnutrition and various chronic diseases that are not transmissible (WHO 2020). Hazelnut is one of the most widely consumed tree nuts (Alasalvar and Shahidi 2009). According to the latest FAO data, the global production of hazelnuts in the hard shell reached approximately 1.13 million tons (FAOSTAT 2025). After the hard shell is removed, the hazelnuts can be consumed with or without skin. Among these, hazelnuts without skin are preferred by consumers. The skin is brown testa that is located under the hard shell of the hazelnut and surrounds the whole kernel. Whilst the hard shell of the hazelnut is broken and easily removed from the hazelnut, the hazelnut skin remains on the surface of the hazelnut kernel. To remove the skin from the hazelnut, a suitable peeling method is needed. The hazelnut skin is peeled using the roasting technique, which is the most widely used and oldest known method. There is even archaeological evidence regarding the roasting process of hazelnuts (Perren and Escher 2013). In fact, the roasting technique is not a special peeling technique developed only to remove the hazelnut skin. It is also used to improve the sensory properties of the hazelnut, such as flavor, color, and texture, to inactivate enzymes, to eliminate microorganisms, and to reduce water activity (Belviso et al. 2017; Marzocchi et al. 2017; Özdemir et al. 2001). The high temperatures applied during roasting loosen the hazelnut skins, allowing them to separate easily from the hazelnut kernels. Therefore, peeling is effectively achieved as a part of the roasting process. Although roasting offers these benefits, it also promotes undesirable reactions such as lipid oxidation at elevated temperatures (Alasalvar et al. 2010; Amaral et al. 2006; Perren and Escher 2013). Lipid oxidation in hazelnuts has been shown to alter fatty acid composition (Alasalvar et al. 2010; Amaral et al. 2006; Kirbaşlar and Erkmen 2003), generate peroxides (Belviso et al. 2017; Özdemir et al. 2001; Özkan et al. 2016) and produce volatile oxidation products (Alasalvar et al. 2003; Marzocchi et al. 2017; Şahin 2023). Roasting also results in losses of essential amino acids (Kirbaslar and Erkmen 2003; Özdemir et al. 2001); phytosterols, tocopherols, and tocotrienols (Amaral et al. 2006); and phenolic acids (Pelvan et al. 2012). Finally, it induces notable changes in the microstructure of the hazelnuts (Alamprese et al. 2009; Perren and Escher 2013).

The pressurized water peeling technique was developed as an alternative to the traditional roasting method. In this approach, hazelnut skins are removed solely by the effect of pressurized water without using any chemicals. After peeling, the wet hazelnuts are centrifuged to eliminate surface water. Unlike roasting, this method does not involve high temperatures; instead, a low‐temperature drying process is applied to remove residual water from the hazelnut surface.

Although some previous studies mentioned above evaluated the chemical composition, microstructure, peroxide value (PV), free fatty acidity (FFA), total antioxidant content (TAC), and total phenolic content (TPC) of hazelnuts peeled by roasting technique, these parameters have not been investigated in hazelnuts peeled using the pressurized water technique during long‐term storage. Therefore, the first goal of this study was to examine the effects of two peeling methods—pressurized water and roasting—on key quality parameters of hazelnuts. Specifically, we assessed proximate composition, fatty acid composition, PV, FFA, TAC, TPC, total aflatoxin content, volatile compounds, and microstructure. The second goal was to evaluate how these quality parameters change over prolonged storage depending on the peeling method. For this purpose, the peeled hazelnuts were stored for 12 months and analyzed at certain periods.

## 2 Materials and Methods

### 2.1 Experimental Materials

Butanol, isooctane, Folin–Ciocalteu, methanol, hexane, and sulfuric acid were acquired from Merck. FAME standard mixture was purchased from Restek. All other chemicals used in the study were supplied by Sigma Aldrich.

### 2.2 Preparation of Hazelnut (*Corylus avellana* L.) Samples

The hazelnut samples used in the study were Tombul hazelnuts supplied from Çelebioğlu Hazelnut Factory (Ordu, Türkiye). In the factory, the hard shells of the hazelnuts were first broken and removed. Then, the hazelnut kernels were taken to the peeling unit of the factory, where the skin of the hazelnut kernels was removed by two different methods: Roasting technique and pressurized water technique. Production with each peeling technique was repeated twice. After the peeling process, hazelnut kernels without skin were stored at room temperature until the storage period of 3, 6, 9, and 12 months. The hazelnut samples that completed their storage period were kept in a frozen state until the analysis.

#### 2.2.1 Roasting Technique (RT)

After hazelnut kernels were roasted at 155°C for 15 min, their skins were removed using a hazelnut peeling machine. The peeled kernels were then stored in 1 kg vacuum packaging.

#### 2.2.2 Pressurized Water Technique (PWT)

In PWT, the skin peeling process was carried out by giving water to hazelnut kernels at a certain pressure (3.8 MPa). Subsequently, hazelnut kernels were centrifuged and dried at 115°C for 45 min, to eliminate any residual water on their surface. Process flow chart and equipment of hazelnut peeling technique with pressurized water were given as Supporting Information (Figures S1 and S2). Hazelnut kernels separated from their skins were then vacuum‐packed in 1 kg packaging for storage.

### 2.3 Analytical Methods

Proximate composition of samples indicates the moisture, ash, oil, and protein content of the peeled hazelnuts. To determine ash content, hazelnut samples were completely burnt at 550°C until light‐gray ash was obtained. Their moisture contents were determined by drying them in an oven at 105°C. Extraction of total oil was carried out for 2 h using n‐hexane with a Soxhlet extraction apparatus. Protein content was determined using the Kjeldahl method, calculated as total nitrogen multiplied by 6.25 (Sahin et al. 2022).

Analysis of fatty acid composition in the peeled hazelnuts was conducted by a GC‐FID (gas chromatography with flame ionization detector) method according to the literature (Şahin and Özata 2022). Briefly, the fatty acids of oil samples were first converted into fatty acid methyl esters (FAMEs). The separation of FAMEs was performed using a GC capillary column (60 m × 0.25 mm x 0.20 µm, TR‐CN100). The oven temperature was initially set at 140°C for 5 min, then increased at a rate of 4°C per minute until reaching 250°C, where it was maintained for 15 min. The injector temperature was set to 250°C, with an injection volume of 1 µL and a split ratio of 1:100. Nitrogen was used as the carrier gas at a flow rate of 30 mL/min. A standard FAME mixture was utilized to identify FAMEs in the oil samples.

To determine free fatty acid (FFA), oil samples extracted from the peeled hazelnuts by cold press were added to a diethyl ether:ethyl alcohol mixture (1:1 volume ratio) and were titrated with sodium hydroxide (0.1 N). Amount of FFA was found in % oleic acid by proportioning the amount of sodium hydroxide spent in titration to the amount of oil sample and multiplying the value by 2.82 (Şahin and Özata 2022).

To determine peroxide value (PV), after oil samples (2–2.5 g) extracted from the peeled hazelnuts by cold press were mixed with isooctane: acetic acid (1:1 volume ratio). Then, potassium iodide (saturated), pure water, and starch solution (1%) were added to mixture. The mixture was titrated with sodium thiosulfate solution (0.1 N) until color disappeared. PV was calculated as meq O_2_/kg based on the amount of sodium thiosulfate spent in titration (Şahin and Özata 2022).

To determine aflatoxins of the peeled hazelnuts, an immunoaffinity column liquid chromatography method as reported by Senyuva and Gilbert (2005) was applied with minor modifications. To condition immunoaffinity column (Vicam AflaTest, USA), the phosphate‐buffered saline (0.1 M pH:7.4) was passed through it. For extraction, 4 g of salt (NaCl), 250 mL of methanol, and 200 mL of water were added to 50 g of ground sample and mixed thoroughly. Then mixture was filtered through filter paper. Resulting filtrate was passed through an immunoaffinity column (Aflaprep, R‐Biopharm Rhône Ltd, Glasgow, Scotland) and subjected to post‐column electrochemical derivatization using Kobra Cell (R‐Biopharm Rhône Ltd, Glasgow, Scotland). Filtrate was injected to an Avantor ACE 5 C18 column (4.6 × 250 mm, 5 µm, VWR International GmbH, Vienna). The HPLC unit (Agilent 1260 Infinity II System, Agilent Technologies, USA) was equipped with a vial sampler (G7129A‐1260), an ISO pump (G7110B‐1260), and a fluorescence light detector (G7121A‐1260). Fluorescence light detection was carried out at an excitation wavelength of 362 nm and an emission of 440 nm. Mobile phase consisted of water and methanol (11:9, v:v). For post‐column electrochemical derivatization, 0.119 mg potassium bromide and 350 µL nitric acid (4 N) were added to each liter of mobile phase.

The volatile substances in the hazelnut samples were analyzed using the HS‐SPME‐GC‐MS method, following the procedure described by Şahin and Topçu (2025) with slight modifications. The separation of volatile compounds was performed using an Rtx‐Wax capillary column (60 m × 0.25 mm × 0.5 µm). The peeled hazelnuts weighing 3 g was placed into vials. SPME fiber (DVB/CAR/PDMS) from Supelco (USA) was introduced into the vial and allowed to equilibrate at 40°C for 30 min. The fiber was then thermally desorbed at 240°C for 7 min. Helium served as carrier gas with a flow rate of 1.5 mL/min. Oven temperature was initially set to 40°C and held for 1 min, then increased to 170°C at a rate of 3°C per minute, maintaining this temperature for 1 min. The oven temperature was programmed as follows: 40°C (held 1 min) to 170°C at 3°C/min (held 1 min) to 240°C at 10°C/min. The ionization voltage was set at 70 eV. Mass spectra were recorded over a range of *m*/*z* 33 to 350 using electron ionization. The identification of volatile compounds in hazelnut samples was conducted through a comparison of the mass spectra of unknown peaks with NIST and FFNSC mass spectral libraries.

Determination of total antioxidant capacity (TAC) in the peeled hazelnuts followed the DPPH assay described by Şahin and Özata (2022). TAC values were expressed in mmol of trolox equivalents (TE) per liter. Total phenol content (TPC) of the peeled hazelnuts was analyzed by Folin–Ciocalteu assay described by Sahin et al. (2022).

According to the method described by Alamprese et al. (2009), surfaces of the peeled hazelnuts were photographed using the scanning electron microscopy (SEM; Hitachi, SU 1510, Japan) under 20 kV voltage and high vacuum. The peeled hazelnuts were sputter‐coated with gold.

### 2.4 Data Analysis

The data was analyzed using repeated measures two‐way ANOVA, followed by Tukey's honest significant difference (HSD) test for multiple comparisons. A significant threshold of *p* < 0.05 was applied. Statistical evaluations were conducted using software (IBMSPSS, version 26)

## 3 Results and Discussion

Limited studies have been published on the chemical composition, fatty acid composition, PV, FFA, TPC, TAC, aflatoxin contents, volatile compounds, and microstructure of hazelnuts peeled using by RT (in another word roasted hazelnuts), to our knowledge, no studies have explored these parameters for hazelnuts peeled using PWT over a prolonged storage period. Therefore, it was not possible to numerically compare our findings for the hazelnuts peeled using PWT with the literature.

Proximate composition (oil, protein, moisture, and ash) of the hazelnut kernels peeled using two different methods‐pressurized water technique (PWT) and roasting technique (RT)—during storage is presented in Table 1. As shown in the table, oil is the main component. The oil content of hazelnuts peeled with RT ranged from 57.24% to 70.57 %. Similar results for roasted hazelnuts were previously reported by Alasalvar et al. (2010), Amaral et al. (2006), Kirbaslar and Erkmen (2003), and Özkan et al. (2016). For hazelnuts peeled using PWT, the oil content ranged from 53.87% to 65.68% (Table 1). Throughout the storage period, oil levels in hazelnuts kernels peeled using PWT were notably lower than those in hazelnuts peeled using RT. This difference is likely due to the elevated temperatures applied during the RT peeling process. As it is known that at higher temperatures, proteins undergo greater denaturation and lipid molecules tend to become more fluid, leading to an increased release of oil from cells. Since a higher temperature (155°C for 15 min) was applied in RT compared to PWT (115°C for 45 min), this high temperature applied in RT may have caused more oil to be extracted from hazelnuts. These findings align well with the results of Alasalvar et al. (2010), who investigated the effect of roasting processes (140°C for 30 min) on oil content of hazelnut varieties and reported that roasting processes increased oil yield of hazelnut varieties. Özkan et al. (2016) also explored the impact of roasting techniques (ranging from 110 to 180°C for 6 to 34 min, employing an experimental design based on central composite design) on the oil characteristics of hazelnuts, and reported that as the roasting temperature and duration increased, so did oil content.

**TABLE 1:** Changes in proximate composition (%) of the hazelnut kernels without skin during storage.

		Duration of storage (in months)				
	Samples	0	3	6	9	12
Oil	WH	53.87 ± 0.00^a,B^	64.39 ± 0.08^c,B^	65.68 ± 0.12^e,B^	65.11 ± 0.02^d,B^	60.65 ± 0.00^b,B^
	RH	57.24 ± 0.04^a,A^	66.45 ± 0.00^b,A^	70.57 ± 0.00^d,A^	69.80 ± 0.05^c,A^	66.60 ± 0.06^b,A^
Moisture	WH	1.89 ± 0.02^b,A^	1.83 ± 0.02^b,A^	1.64 ± 0.00^a,A^	2.04 ± 0.04^c,A^	2.16 ± 0.01^c,A^
	RH	1.51 ± 0.41^b,B^	1.25 ± 0.01^a,B^	1.38 ± 0.08^ab,B^	1.87 ± 0.04^c,B^	1.88 ± 0.07^c,B^
Protein	WH	16.39 ± 0.49^b,B^	17.49 ± 0.08^bc,B^	14.61 ± 0.03^a,B^	18.07 ± 0.13^c,A^	20.51 ± 0.07^d,A^
	RH	18.32 ± 0.11^d,A^	19.96 ± 0.05^e,A^	17.58 ± 0.08^c,A^	14.97 ± 0.16^a,B^	16.57 ± 0.08^b,B^
Ash	WH	2.05 ± 0.01^a,A^	2.09 ± 0.02^a,A^	2.10 ± 0.21^a,A^	2.07 ± 0.18^a,A^	2.08 ± 0.03 ^a,A^
	RH	2.06 ± 0.04^a,A^	2.06 ± 0.05^a,A^	2.03 ± 0.06^a,A^	2.00 ± 0.01^a,A^	1.96 ± 0.08^a,A^

*Note*: The values presented represent means along with their respective standard deviations. Distinct capital letters within the same columns or by different lower‐case letters within the same rows indicated statistically significant differences (*p* ≤ 0.05).

Abbreviations: RH, hazelnut kernels, whose skins were removed by RT; WH, hazelnut kernels, whose skins were removed by PWT.

Over the storage period, the oil content of hazelnuts peeled using both techniques fluctuated but tended to increase slightly overall. These changes may be related to fluctuations in moisture content of the peeled hazelnuts during storage. This trend is consistent with the findings of Turan and Karaosmanoğlu (2019), who reported similar fluctuations and an overall increase in oil content in hazelnuts (in‐shell, unroasted) stored for 2 years.

Moisture values of the hazelnuts peeled with RT varied from 1.25% to 1.88%, while the hazelnuts peeled with PWT had moisture ranging from 1.64% to 2.16% (Table 1). During the 12‐month storage period, hazelnuts peeled using PWT had significantly higher moisture content than those peeled using RT. This is an expected result and indicates that more water evaporates from the hazelnuts due to the high temperature applied during RT. The result is supported by Amaral et al. (2006) and Marzocchi et al. (2017), who investigated effects of different roasting conditions (temperature and time) on physical–chemical properties of hazelnuts and found that water loss increased with higher roasting temperatures and longer durations. Furthermore, the moisture contents of hazelnuts peeled with both techniques increased slightly but significantly after 12‐month storage (Table 1). Similarly, Ghirardello et al. (2013) observed a significant increase in the moisture content of unroasted hazelnuts stored at room temperature after 12‐months storage.

During storage, the protein contents of hazelnuts peeled with PWT and hazelnuts peeled with RT were 14.61%–20.51% and 14.97%–19.96%, respectively. Hazelnuts peeled using PWT exhibited significantly lower protein content before storage compared to hazelnuts peeled using RT. Because previous studies demonstrated that the protein content of hazelnuts is not significantly influenced by the temperature applied during RT (Kirbaşlar and Erkmen 2003; Özdemir et al. 2001), the observed difference is more likely due to the loss of water‐soluble proteins caused by the pressurized water applied during PWT. Throughout storage, there were fluctuations in protein content in hazelnuts peeled with both techniques. Notably, from the 9th month onward, protein levels in PWT‑peeled hazelnuts increased markedly. Consistent with our findings, Bostan and Güler (2016) and Akar and Bostan (2018) reported that the protein content of various hazelnut varieties (in‐shell, unroasted) fluctuated during storage and showed a significant increase by the 9th month. In contrast, hazelnuts peeled with RT experienced a pronounced protein decline in the 9th month. We hypothesize that these divergent trends reflect differences in the predominant protein fractions: Storage may promote the accumulation of proteins more resistant to water‐based processing in hazelnuts peeled with PWT samples, whereas the hazelnuts peeled with RT lose labile proteins over time. Such behavior—where certain proteins increase and others decrease during prolonged storage—has been documented in other foods, including milk powder and rice (Fyfe et al. 2011; Wongdechsarekul and Kongkiattikajorn 2010; Zhao et al. 2021). These compositional shifts correspond to structural changes at the molecular level: storage induces the breakdown of α‑helix and β‑sheet domains and fosters the formation of β‑turns and random coils (Zhao et al. 2021).

According to the ash results presented in Table 1, it was found that the amounts of ash in the hazelnuts were not significantly affected by peeling technique. Moreover, no significant change was detected in the amount of ash depending on the storage period.

Hazelnut oil, abundant in unsaturated fatty acids, primarily contains monounsaturated oleic acid. This is followed by polyunsaturated linoleic acid, saturated palmitic acid, and saturated stearic acid (Crews et al. 2005; Schlörmann et al. 2015; Sahin et al. 2022; Şahin 2023). Alterations in major fatty acid profiles of the peeled hazelnuts during storage are shown in Table 2. The amounts of palmitic acid in hazelnuts peeled with PWT and in hazelnuts peeled with RT were found to be 4.49% to 4.61% and 4.33% to 4.81%, respectively. These values are similar to the values for roasted hazelnuts reported by Amaral et al. (2006) and by Schlörmann et al. (2015), but lower than the values found by Alasalvar et al. (2010) and Crews et al. (2005). According to statistical analyses, peeling techniques (PWT and RT) did not significantly affect palmitic acid contents in hazelnuts. Stearic acid content in hazelnuts peeled using PWT varied from 2.10% to 2.33%, whereas in hazelnuts peeled using RT, it varied from 2.11% to 2.34%. Stearic acid values for hazelnuts peeled using RT were in agreement with previous studies evaluating fatty acid composition in hazelnuts roasted at different temperatures (Alasalvar et al. 2010; Amaral et al. 2006; Crews et al. 2005; Schlörmann et al. 2015). A significant difference in the stearic acid content of the hazelnuts depending on the peeling technique was observed only at the beginning (0th month) and 6th month of storage. During storage, oleic acid contents in hazelnuts peeled with PWT and hazelnuts peeled with RT were determined to be in the range of 83.94%–84.34% and 83.65%–84.09 %, respectively. Similar results for the oleic acid content of roasted hazelnuts have been reported by Alasalvar et al. (2010), Amaral et al. (2006), Crews et al. (2005), Kirbaşlar and Erkmen (2003), and Schlörmann et al. (2015). Furthermore, no significant difference was observed in the oleic acid content of hazelnuts depending on peeling technique throughout storage. Linoleic acid content in hazelnuts peeled with PWT ranged from 7.14% to 8.26%, whereas in hazelnuts peeled with RT, it ranged from 7.55% to 8.42%. Similar linoleic acid content was reported by Alasalvar et al. (2010) for some roasted hazelnut varieties and by Schlörmann et al. (2015) for roasted hazelnuts. On the other hand, some researchers have reported higher linoleic acid levels in roasted hazelnuts (Amaral et al. 2006; Kirbaşlar and Erkmen 2003). As can be seen in Table 2, there was no significant difference between hazelnuts peeled using PWT and hazelnuts peeled using RT until 9th month of storage. However, after 12‐month storage, a small but significant difference was observed in linoleic acid content of hazelnuts peeled with PWT and hazelnuts peeled with RT.

**TABLE 2:** Alterations in fatty acid profiles of the hazelnut kernels without skin during storage.

		Duration of storage (in months)				
	Samples	0	3	6	9	12
Palmitic acid [%]	WH	4.53 ± 0.32^a,A^	4.52 ± 1.13^a,A^	4.61 ± 0.06^a,A^	4.54 ± 0.04^a,A^	4.49 ± 0.01^a,A^
	RH	4.33 ± 0.06^a,A^	4.56 ± 0.03^a,A^	4.81 ± 0.09^a,A^	4.64 ± 0.15^a,A^	4.59 ± 0.03^a,A^
Stearic acid [%]	WH	2.10 ± 0.01^a,B^	2.15 ± 0.02^a,A^	2.23 ± 0.00^a,B^	2.33 ± 0.17^a,A^	2.29 ± 0.01^a,A^
	RH	2.34 ± 0.02^a,A^	2.11 ± 0,00^a,A^	2.29 ± 0.00^a,A^	2.18 ± 0.14^a,A^	2.24 ± 0.03^a,A^
Oleic acid [%]	WH	84.31 ± 0.46^a,A^	83.95 ± 0.12^a,A^	84.34 ± 0.04^a,A^	84.19 ± 0.05^a,A^	83.94 ± 0.34^a,A^
	RH	84.09 ± 0.22^a,A^	83.76 ± 0.12^a,A^	83.99 ± 0.11^a,A^	83.99 ± 0.29^a,A^	83.65 ± 0.03^a,A^
Linoleic acid [%]	WH	8.26 ± 0.16^c,A^	7.95 ± 0.04^bc,A^	7.51 ± 0.10^ab,A^	7.49 ± 0.07^ab,A^	7.14 ± 0.01^a,B^
	RH	8.42 ± 0.10^b,A^	7.93 ± 0.02^ab,A^	7.55 ± 0.05^a,A^	7.65 ± 0.19^a,A^	8.11 ± 0.04^ab,A^

*Note*: The values presented represent means along with their respective standard deviations. Distinct capital letters within the same columns or by different lower‐case letters within the same rows indicated statistically significant differences (*p* ≤ 0.05).

Abbreviations: RH, hazelnut kernels, whose skins were removed by RT; WH, hazelnut kernels, whose skins were removed by PWT.

Among these major fatty acids mentioned above, only a change in the amount of linoleic acid was observed depending on storage time. During storage, the amount of linoleic acid in the hazelnuts peeled with PWT significantly decreased. Whilst a similar decrease in linoleic acid content was observed in hazelnuts peeled using RT until the 6 th month of storage, no significant change in linoleic acid was observed after 6^‐^ th month of storage. The decrease in linoleic acid content with increasing storage time is an expected result and is attributed to oxidation. This is because linoleic acid due to the two double bonds in its structure (18:2(9,12)) undergoes more oxidation compared to saturated palmitic (16:0) and stearic (18:0) acids and monounsaturated oleic acid (18:1(9)) (Amaral et al. 2006; Kirbaşlar and Erkmen 2003; Şahin 2023).

As it is known, FFA is a measure of the degradation of fats via hydrolysis. Hazelnuts peeled with PWT had similar FFA values to the hazelnuts peeled using RT in all storage periods (*p* > 0.05) except the 6th month of storage, in which hazelnuts peeled using PWT exhibited a significantly higher FFA (Table 3). The higher FFA value observed in hazelnuts peeled with PWT may be related to the fact that the water used in the peeling process causes more hydrolysis in the oils. Another possible reason for higher FFA content in hazelnuts peeled using PWT compared to hazelnuts peeled by RT is the inactivation of hydrolytic enzymes at high temperatures used in roasting. Özdemir et al. (2001) recorded that FFA in hazelnuts decreased when roasting temperatures exceeded 120°C, attributing this to enzyme inactivation caused by heat. Additionally, during the 12‐month storage period, a more pronounced increase in FFA was observed in hazelnuts peeled with PWT (1.8‐fold) compared to hazelnuts peeled by RT (1.2‐fold). This increase in FFA values in hazelnuts during storage is not unexpected. A similar trend was reported by Ghirardello et al. (2013), who observed rising FFA levels in hazelnuts with skin stored under various conditions for twelve months.

**TABLE 3:** Changes in FFA (%), PV (meq O_2_/kg), TFC (mmol GAE/L), and TAC (mmol TE /L) of the hazelnut kernels without skin during storage.

		Duration of storage (in months)				
	Samples	0	3	6	9	12
FFA [%]	WH	0.31 ± 0.01^ab,A^	0.29 ± 0.02^a,A^	0.46 ± 0.01^c,A^	0.39 ± 0.01^bc,A^	0.56 ± 0.01^d,A^
	RH	0.38 ± 0.03^ab,A^	0.28 ± 0.04^a,A^	0.34 ± 0.00^ab,B^	0.31 ± 0.01^ab,A^	0.46 ± 0.02^b,A^
PV [meq O_2_/kg]	WH	0.00 ± 0.00^a,A^	0.22 ± 0.02^ab,A^	0.22 ± 0.02^ab,A^	0.24 ± 0.09^ab,A^	0.29 ± 0.04^b,A^
	RH	0.92 ± 0.00^a,B^	0.93 ± 0.00^a,B^	1.39 ± 0.05^a,B^	2.15 ± 0.18^b,B^	2.38 ± 0.03^b,B^
TFC [mmol GAE/L]	WH	1.87 ± 0.03^b,A^	1.87 ± 0.02^b,A^	1.32 ± 0.06^a,A^	1.12 ± 0.06^a,A^	1.01 ± 0.11^a,A^
	RH	1.72 ± 0.00^c,B^	1.56 ± 0.01^bc,B^	1.27 ± 0.17^ab,A^	0.94 ± 0.00^a,A^	0.87 ± 0.04^a,A^
TAC [mmol TE/L]	WH	0.58 ± 0.04^b,A^	0.49 ± 0.01^ab,A^	0.44 ± 0.00^a,A^	0.42 ± 0.01^a,A^	0.41 ± 0.00^a,A^
	RH	0.42 ± 0.00^a,A^	0.36 ± 0.01^a,B^	0.35 ± 0.06^a,A^	0.32 ± 0.07^a,A^	0.28 ± 0.03^a,A^

*Note*: The values presented represent means along with their respective standard deviations. Distinct capital letters within the same columns or by different lower‐case letters within the same rows indicated statistically significant differences (*p* ≤ 0.05).

Abbreviations: FFA, free fatty acids; PV, peroxide number; RH, hazelnut kernels, whose skins were removed by RT; TAC, total antioxidant capacity; TFC, total phenolic content; WH, hazelnut kernels, whose skins were removed by PWT.

Peroxide value (PV) indicates the level of lipid oxidation. During storage, changes of PV in hazelnuts peeled by PWT and RT are listed in Table 3. At the beginning of storage, no peroxide formation was detected in hazelnuts peeled using PWT (PV = 0.00 meq O_2_/kg), whereas the PV in hazelnuts peeled using RT was 0.92 meq O_2_/kg. Throughout the storage period, hazelnuts peeled with RT consistently exhibited significantly higher PV values than hazelnuts peeled with PWT. By the end of storage, the PV in hazelnuts peeled with RT was 8.2 times higher than that of hazelnuts peeled with PWT. This difference may be attributed to the high temperatures used in RT, which promote greater lipid peroxidation. These findings are consistent with previous studies (Belviso et al. 2017; Özkan et al. 2016), which reported that higher roasting temperatures further increased PV in hazelnuts. As expected, PV also increased over time in hazelnuts peeled with both techniques. Similar trends were observed by Ghirardello et al. (2013) for unroasted hazelnuts stored for 12 months and by Belviso et al. (2017) for roasted hazelnuts stored for 9 months.

As can be seen in Table 3, during 12 months of storage, TFC in the hazelnuts peeled with PWT decreased from 1.87 mmol GAE/L to 1.01 mmol GAE/L, whereas a decrease in the TFC of the hazelnuts peeled with RT from 1.72 mmol GAE/L to 0.87 mmol GAE/L was detected. Contrary to this decrease observed here, depending on storage time, Belviso et al. (2017) did not detect a regular decrease depending on storage time in hazelnuts, which were roasted at different conditions (120°C for 40 min and 170°C for 20 min) and stored for 9 months. During storage, hazelnuts peeled with PWT exhibited higher TFC compared with hazelnuts peeled using RT, and this difference between them was found to be statistically significant only at the beginning and the 3^‐^ rd month of storage. These differences in TPC observed in hazelnuts herein were likely due to degradation of some phenolics during high‐temperature roasting, as previously reported by other authors (Schmitzer et al. 2010; Pelvan et al. 2012). In contrast, Belviso et al. (2017) and Marzocchi et al. (2017) recorded that roasting treatment led to an increase in TPC of hazelnuts.

According to results presented in Table 3, a significant decline in TAC of the hazelnuts peeled with PWT from 0.58 mmol TE/L to 0.41 mmol TE/L was determined during 12 months of storage, while TAC in hazelnuts peeled with RT did not change significantly and varied between 0.28 mmol TE/L and 0.42 mmol TE/L. The hazelnuts peeled with PWT showed higher TAC than hazelnuts peeled using RT in all storage periods, but the only significant difference between the TAC values of the hazelnuts was detected in the 3^‐^ rd month of storage. These results are consistent with the TFC results given above. The reason for higher TAC in hazelnuts peeled with PWT may be that some effective antioxidant compounds of the hazelnuts break down and disappear at the high temperature applied in RT. These results are supported by Schlörmann et al. (2015), who investigated the effect of roasting conditions (123°C to 185.5°C for maximum 25 min) on TAC, health‐related compounds, and lipid oxidation in different nuts and recorded that the roasting treatment at higher temperatures led to a decrease of TAC in hazelnuts. Similarly, Pelvan et al. (2012) reported that roasting treatment led to ‐a loss in TAC of hazelnuts. On the other hand, Belviso et al. (2017) recorded that high temperature roasting process generally increased TAC in some types of hazelnuts but decreased it in some types of hazelnuts (Ordu), depending on roasting temperature and roasting time.

The presence of aflatoxins, including B1, B2, G1, and G2 forms, is one of the biggest problems in hazelnut production, processing, storage, and export. They are mycotoxins produced by fungi of the genus *Aspergillus*. Of these, B1 and B2 forms are produced by *A. flavus*, while G1 and G2 forms are generated by *A. parasiticus* (Prelle et al. 2012). According to European Commission Regulation 2010/165/EC (European Commission, 2010), the legal limit of total in hazelnut for direct human consumption is 10 µg/kg to ensure food safety. The total amounts of aflatoxins (B1 + B2 + G1 + G2) in hazelnuts peeled with different techniques are illustrated in Table 4. According to the results in Table 4, the total aflatoxin content of hazelnuts was determined to be below 1 µg/kg. No statistically significant difference was found herein depending on both storage time and peeling technique. The total amounts of aflatoxins in hazelnuts peeled with PWT were measured as 0.60 µg/kg at the beginning of storage and 0.52 µg/kg after 12 months of storage. In hazelnuts peeled with RT, the total aflatoxin content was calculated as 0.77 µg/kg at the beginning of storage and 0.54 µg/kg after 12 months of storage. Similarly, Prelle et al. (2012) detected a total aflatoxin amount for roasted hazelnuts as 0.62 µg/kg. Baltaci et al. (2012) determined the total aflatoxin amounts in roasted hazelnuts in the range of 0.07–43.59 µg/kg.

**TABLE 4:** Changes in total aflatoxin contents (µg/kg) of the hazelnut kernels without skin during storage.

	Duration of storage (in months)	
Samples	0	12
WH	0.60 ± 0.01	0.52 ± 0.06
RH	0.77 ± 0.07	0.54 ± 0.03

*Note*: The values presented represent means along with their respective standard deviations.

Abbreviations: RH, hazelnut kernels, whose skins were removed by RT; WH, hazelnut kernels, whose skins were removed by PWT.

Primary volatile compounds detected in the hazelnuts peeled with two different techniques (PWT and RT) at the beginning and after storage of 12 months are listed in Table 5. According to Table 5, it is seen that most of the hazelnut volatile compounds are fat‐derived volatile compounds such as fatty alcohols, fatty acids, and fatty acid esters. This may be because the composition of hazelnuts consists mostly of oil. The volatile compounds detected in the hazelnuts peeled with RT are consistent with previously reported findings by Alasalvar et al. (2003), Artik et al. (2021), and Marzocchi et al. (2017), who identified volatile aroma components of roasted hazelnuts. When the volatile components of hazelnuts peeled with PWT were compared with the volatile components of hazelnuts peeled with RT, propylene glycol; pinacol; 2‐ethoxy ethanol; furfuryl alcohol; 1‐butanol; 1‐pentanol; 1‐hexanol; 2‐ethyl hexanol, 4‐methyl 2‐pentanol, pentanal, hexanal, octanal, methyl propenyl ketone, 5‐methyl‐2‐hepten‐4‐one, hydroxy acetone, butyrolactone, ethanoic acid, ethyl 3‐hydroxybutyrate, and hexanoic acid were detected in both peeled hazelnuts (not stored). Among these volatile compounds, 5‐methyl‐2‐hepten‐4‐one has been identified in previous studies as filbertone (characteristic hazelnut‐like aroma) and an important flavor compound found the hazelnuts (Alasalvar et al. 2003; Artik et al. 2021; Booth et al. 2024; Marzocchi et al. 2017). Unlike the hazelnuts peeled with RT (not stored), 10 volatile compounds including four alcohols (1‐heptanol, 3‐methyl‐2‐butanol, 1,3,4‐trimethoxy‐2‐butanol and 3,6,9,12,15‐pentaoxanonadecan‐1‐ol), 1 aldehyde (metaldehyde isomer II), 1 acid (butyl lactate), two esters (butyl butanoate and butyl isobutyrate), and two amines (2‐butylamine and pyrrole) were found in hazelnuts peeled with PWT (not stored). Seventeen distinct volatile compounds including two alcohols (2‐pentanol and 2‐phenyl‐2 propanol), four aldehydes (furfural, benzaldehyde, paracetaldehyde isomer I and metaldehyde isomer I), three ketones (2‐methyl‐heptan‐3‐one, acetoin and undecan‐2‐one), three acids (methanethiol butyrate, 2‐hydroxy‐4‐methyl pentanoate and hexyl lactate), two pyrazines (2‐methyl pyrazine and 2,5‐dimethyl pyrazine), one amine (pentylamine), and two ethers (diethyl acetal and isopropyl phenethyl ether) were identified in the hazelnuts peeled with RT (not stored) that are not present in the hazelnuts peeled with PWT (not stored). This indicates that more volatile compounds, especially aldehydes, ketones, and pyrazines, were formed in the hazelnuts during RT compared to PWT. These results are consistent with previous findings by Alasalvar et al. (2003), Artik et al. (2021), and Marzocchi et al. (2017). The authors reported that the roasting process is responsible for the formation of more volatile compounds in the hazelnuts. It has also been reported that some of these compounds are formed because of the oxidation of lipids during the roasting process. In addition, it has been reported that some of these compounds (aldehydes and ketones) are released by the oxidation of lipids during roasting process, while some of them such as pyrazines are formed by the Maillard reaction that occurs between sugars and amino acids during roasting (Alasalvar et al. 2003; Perren and Escher 2013).

**TABLE 5:** Primary volatile substances detected in the hazelnut kernels without skin during storage, with their corresponding peak areas identified.

			Area of peak × 10^3^		
Retention index	Volatile compounds	WH month 0	RH month 0	WH month 12	RH month 12
	**1. Alcohols**				
	**1.1. Glycols**				
938	Propylene glycol	131	70	100	64
1619	Pinacol	58	86	*	*
1660	2‐Ethoxy ethanol	69	138	*	*
	**1.2. Fatty alcohols**				
679	3‐Butyn‐1‐ol	*	*	*	96
1148	1‐Butanol	42	86	*	100
1255	1‐Pentanol	81	91	344	1715
1358	1‐Hexanol	78	96	307	124
1461	1‐Heptanol	55	*	214	481
1495	2‐Ethyl hexanol	196	302	141	175
1560	2‐Pentanol	*	120	*	*
1565	1‐Octanol	*	*	53	198
1614	4‐Methyl 2‐pentanol	60	74	36	*
1621	Heptan‐3‐ol	*	*	29	*
1790	Heptan‐2‐ol	*	*	*	96
	**1.3. Other alcohols (primary, secondary, aliphatic, aromatic, etc.)**				
1123	3‐Methyl‐2‐butanol	34	*	*	*
1158	Oct‐3‐en‐2‐ol	*	*	41	199
1640	2‐Phenyl‐2 propanol	*	136	*	*
1669	Furfuryl alcohol	54	73	*	*
1718	1,3,4‐Trimethoxy‐2‐butanol	75	*	*	*
1807	3,6,9,12,15‐Pentaoxanonadecan‐1‐ol	1297	*	*	*
	**2. Aldehydes**				
988	Pentanal	118	110	293	1147
1092	Hexanal	226	1616	2971	17358
1197	Heptanal	*	*	169	360
1302	Octanal	35	68	300	995
1410	Nonanal	*	*	*	452
1449	(*E*)‐2‐octenal	*	*	*	370
1481	Furfural	*	123	*	96
1553	Benzaldehyde	*	82	*	*
1712	Paracetaldehyde isomer I	*	72	*	*
1829	Metaldehyde isomer I	*	98	*	*
1848	Metaldehyde isomer II	59	*	*	*
	**3. Ketones**				
819	Acetone	*	*	46	100
1098	2‐Methyl‐heptan‐3‐one	*	92	*	*
1138	Methyl propenyl ketone	206	433	*	41
1194	Heptan‐2‐one	*	*	56	109
1307	5‐Methyl‐2‐hepten‐4‐one	67	90	126	248
1318	Hydroxyacetone	87	319	*	199
1597	Acetoin	*	60	*	*
1686	Octan‐3‐one	*	*	16	362
1664	Butyrolactone	61	107	*	129
1778	Undecan‐2‐one	*	89	*	*
	**4. Acids**				
	**4.1. Fatty acids**				
832	Methyl acetate	*	*	22	*
1044	Ethyl butyrate	*	*	45	60
1456	Ethanoic acid	662	1007	1121	2489
1502	Methanethiol butyrate	*	61	*	*
1744	Pentanoic acid	*	*	47	416
1764	2‐Hydroxy‐4‐methyl pentanoate	*	85	67	*
1788	Ethyl 3‐hydroxybutyrate	78	88	*	*
1853	Hexanoic acid	205	474	289	2250
	**4.2. Carboxylic acids**				
1769	Hexyl lactate	*	151	34	*
1795	Butyl lactate	150	*	*	*
	**5. Esters**				
	**5.1. Fatty acid esters**				
1700	Butyl butanoate	129	*	*	*
1846	Propyl butanoate	*	*	27	*
	**5.2. Carboxylic esters**				
1798	Butyl isobutyrate	416	*	*	*
	**6. Pyrazines**				
1285	2‐Methyl pyrazine	*	77	*	56
1343	2,5‐Dimethyl pyrazine	*	183	*	461
	**7. Hydrocarbons**				
739	Heptane	*	*	56	271
800	Octane	*	*	127	481
	**8. Amines**				
682	2‐Butylamine	71	*	164	109
1013	Pyrrole	51	*	44	*
1715	Pentylamine	*	70	*	107
	**9. Ethers**				
1523	Diethyl acetal	*	77	*	*
1634	Isopropyl phenethyl ether	*	99	*	*
					

Abbreviations: *, not detectable; RH, hazelnut kernels, whose skins were removed by RT; WH, hazelnut kernels, whose skins were removed by PWT.

**FIGURE 1:**
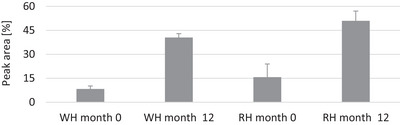
Changes in hexanal contents (%) of the hazelnut kernels without skin during storage. The values presented represent means along with their respective standard deviations. Abbreviations: RH, hazelnut kernels, whose skins were removed by RT; WH, hazelnut kernels, whose skins were removed by PWT.

When hazelnut peeled samples stored for 12 months are compared with not‐stored hazelnut peeled samples, it is seen that 17 of the volatile compounds given in Table 5 are detected in the hazelnut samples after storage. Among them, 1‐octanol, heptanal, acetone, heptan‐2‐one, octan‐3‐one, ethyl butyrate, pentanoic acid, heptane, octane, and oct‐3‐en‐2‐ol were found in both peeled hazelnuts after storage of 12 months. Additionally, 3‐butyn‐1‐ol, heptan‐2‐ol, nonanal and (*E*)‐2‐octenal were detected only in the hazelnuts peeled with RT (after storage), whereas heptan‐3‐ol, methyl acetate and propyl butanoate were identified only in the hazelnuts peeled with PWT (after storage). Some volatile components, including 1‐pentanol, 1‐hexanol, pentanal, hexanal, octanal, 5‐methyl‐2‐hepten‐4‐one, ethanoic, and hexanoic acids, were detected in hazelnut samples both before and after storage, and the peaks representing these compounds were found to have larger areas after storage. These volatile compounds, which are mostly acid, alcohol, aldehyde, and ketone in character, may have been formed more by hydrolytic or oxidative deterioration of lipids in the hazelnuts during storage. It has been reported that some of these volatile compounds such as pentanal, hexanal, octanal, (*E*)‐2‐octenal and nonanal are produced by lipid oxidation and are the most abundant volatile oxidation products of vegetable oils (Mildner‐Szkudlarz et al. 2003; Petersen et al. 2012a and Petersen et al. 2012b; Şahin 2023, Şahin and Topçu 2025). Of them especially hexanal, a secondary reaction product, is used in the monitoring of lipid oxidation (Petersen et al. 2012a; Perren and Escher 2013). The change in the hexanal content due to storage is shown in Figure 1. It was here determined that the hexanal content of hazelnuts peeled with RT was 1.9 times higher than the hazelnuts peeled with PWT, and also that the hexanal content increased for both hazelnuts during storage. This result indicates that the hazelnuts peeled by RT at high temperatures are exposed to more oxidation and that oxidation increases with storage. These results are also compatible with the peroxide results showing lipid oxidation given in Table 3.

**FIGURE 2:**
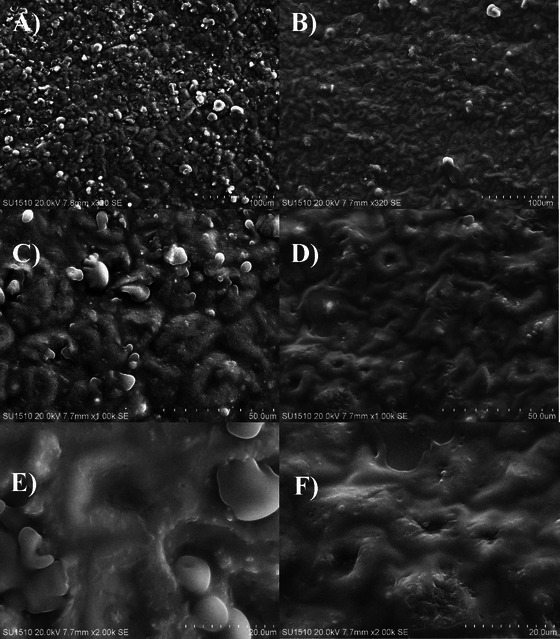
SEM images of the surfaces of the hazelnuts peeled with RT (A, C, and E) and the hazelnuts peeled with PWT (B, D, and F). Scale on zoomed in images: 100 µm for (A) and (B); 50 µm for (C) and (D); and 20 µm for (E) and (F).

Microstructures of the hazelnuts peeled with different methods are given in Figure 2. As in other nuts, oleosomes are prominent in the microstructure of the hazelnuts due to their high oil content (Alamprese et al. 2009; Perren and Escher 2013). The oleosomes are oil stores in the cells and are surrounded by a protective membrane consisting of phospholipids and proteins (Abdullah et al. 2020; Nikiforidis 2019). The SEM images here showed that there were more burst oleosomes on the surface of the hazelnuts peeled with RT compared to the hazelnuts peeled with PWT. The reason for more burst oleosomes in the hazelnuts peeled with RT is probably due to the high temperature applied in RT. Indeed, it has been reported that the roasting temperature affects the microstructure of the hazelnuts (Alamprese et al. 2009; Perren and Escher 2013; Saklar et al. 2003). The high temperature completely destroys the endoplasmic network separating the oleosomes from each other, disrupts proteins in the membrane of the oleosomes, and increases the diameter of the oleosomes. Thus, high temperatures lead to an increase in intercellular fat flow, oxygen transfer, and subsequent lipid oxidation (Abdullah et al. 2020; Alamprese et al. 2009; Perren and Escher 2013; Saklar et al. 2003). This result observed here is in line with the oil results given in Table 1 above, where it was determined that the hazelnuts peeled with RT contained more oil than the hazelnuts peeled with PWT. Additionally, this result is also consistent with the peroxide results in Table 3 and with the hexanal content in Figure 1, such that the hazelnuts peeled with RT were more oxidized than the hazelnuts peeled with PWT. In zoomed SEM images (50.0 and 20 µm, Figure 2C–F), there appear to be more epidermal cell collapses on the surface of the hazelnuts peeled with PWT compared to the hazelnuts peeled with RT. Due to the pressurized water used in the peeling technique, the surface of the hazelnuts may have been further damaged and collapsed.

## 4 Conclusion

In this study, we evaluated the effect of the new peeling method PWT on proximate composition, fatty acid composition, ultrastructure, and other quality parameters, including PV, FFA, TAC, TPC, total aflatoxin content, and volatile compounds of hazelnut samples during 12 months of storage. Additionally, the effect of PWT on these parameters in hazelnut was compared with RT. In terms of oxidation parameters, the hazelnuts peeled by RT exhibited more peroxide formation, more volatile lipid oxidation products, and more hexanal content during storage. In addition, it was observed by SEM that oleosomes were more burst in the microstructure of the hazelnuts peeled by RT. In conclusion, these results indicate that the hazelnuts peeled by RT were exposed to more oxidation, probably because of the high temperature which applied during the roasting. Therefore, in terms of healthy nutrition, PWT is recommended as a hazelnut peeling method due to its ability to reduce oxidation and better preserve hazelnut quality during storage.

## Author Contributions

**Sümeyye Şahin**: conceptualization, investigation, funding acquisition, writing – original draft, methodology, visualization, writing – review and editing, software, project administration, resources, supervision, data curation, validation. **Yeşim Aydın**: formal analysis, visualization, methodology, resources, validation, software.

## Conflicts of Interest

The authors declare no conflicts of interest.

## Supporting information




**Supplementary Material**: Figure jfds70384‐Sup‐0001‐figure.docx
