# Supplementary material for: Increasing the Oxidation Stability and Shelf‐Life Quality of Hazelnuts Using a New Peeling Technique
Source: J Food Sci. 2025 Jul 3;90(7):e70384. doi: 10.1111/1750-3841.70384 (PMC12226203; doi:10.1111/1750-3841.70384)
Supplement: Supplementary file 1 — Supplementary Material: Figure jfds70384‐Sup‐0001‐figure.docx [file JFDS-90-0-s001.docx]

Vibratory Inlet Bunker

Input Elevator (Z Elevator)

Hazelnut Shelling Machine with Pressurized Water (3.8 MPa)

Modular Elevator

Pneumatic Return Bunker

Centrifuge

Vibratory Inlet Bunker

Elevator

Drying Oven (115°C for 45 min)

Output Elevator

**Figure S1** Process flow chart of hazelnut peeling technique with pressurized water


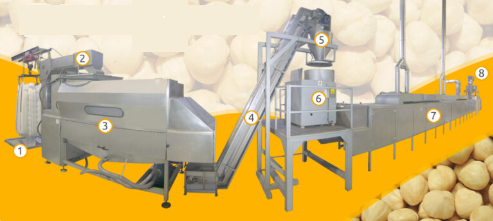


**Figure S2** Equipments of hazelnut peeling technique with pressurized water (https://www.aynut.com.tr/su-ile-findik-zar-soyma-sistemi/
